# Determinants of vascular structure and function in at-risk children born to mothers managed for pre-eclampsia (FINNCARE study)

**DOI:** 10.3389/fcvm.2023.1264921

**Published:** 2023-10-04

**Authors:** Michelle Renlund, Tiina Jääskeläinen, Anni Kivelä, Seppo Heinonen, Hannele Laivuori, Taisto Sarkola

**Affiliations:** ^1^ Children’s Hospital, University of Helsinki and Helsinki University Hospital, Helsinki, Finland; ^2^ Minerva Foundation Institute for Medical Research, Helsinki, Finland; ^3^ Medical and Clinical Genetics, University of Helsinki and Helsinki University Hospital, Helsinki, Finland; ^4^ Department of Food and Nutrition, University of Helsinki, Helsinki, Finland; ^5^ Department of Obstetrics and Gynecology, Helsinki University Hospital, Helsinki, Finland; ^6^ Department of Obstetrics and Gynecology, Tampere University Hospital and Tampere University, Tampere, Finland; ^7^ Faculty of Medicine and Health Technology, Tampere Center for Child, Adolescent, and Maternal Health Research, Tampere, Finland

**Keywords:** pre-eclampsia, adventitia thickness, intima-media thickness, carotid stiffness, blood pressure, ultra-high-frequency ultrasound, cardiovascular disease, arterial health

## Abstract

**Background and aim:**

Pre-eclampsia (PE) is related to elevated blood pressure (BP) in children. The study aims to investigate if elevated BP is reflected in child arterial health and how anthropometrics, body composition, and gestational and perinatal factors influenced this.

**Methods:**

In this prospective cohort study, we assessed the arteries of 182 children exposed (46 had an early onset, with a diagnosis before 34 gestational weeks, and 136 had a late onset) and 85 children unexposed (non-PE) to PE at 8–12 years from delivery using ultra-high-frequency ultrasound in addition to ambulatory and central BPs, body composition and anthropometrics, and tonometry-derived pulse wave velocity (PWV).

**Results:**

No differences were found in intima-media thickness (IMT), adventitia thickness (AT), lumen diameter (LD), local carotid artery stiffness, distensibility, or wall stress between PE-exposed and non-PE-exposed children. All children's brachial, radial, and femoral artery IMTs were associated with 24-h systolic BP (SBP) and pulse pressure, carotid–femoral PWV, and anthropometric measures. The 24-h SBP and anthropometrics, notably lean body mass, were independent predictors of peripheral artery IMTs (brachial *R*^2 ^= 0.217, radial *R*^2 ^= 0.208, femoral *R*^2 ^= 0.214; *p* < 0.001). Head circumference predicted carotid artery IMT and LD (*β* = 0.163, *p* = 0.009; *β* = 0.417, *p* < 0.001, respectively), but carotid artery IMT was not associated with BP. No independent associations were found for peripheral artery ATs. Local carotid artery stiffness, distensibility, and wall stress were independently associated with adiposity. No significant associations were found between gestational or perinatal factors and child vascular health parameters.

**Conclusions:**

The peripheral artery IMT of PE-exposed children is identical to that of non-PE-exposed children, but associated with BP. Adiposity is related to local carotid artery stiffness. These adverse associations in arterial health may reflect the early progression of cardiovascular disease in PE-exposed children.

## 1. Introduction

Pre-eclampsia (PE) affects 2%–8% of pregnancies (1) and is defined as gestational hypertension with new-onset proteinuria, maternal organ dysfunction, and/or uteroplacental dysfunction at 20 weeks of gestation or after (2). Atherosclerotic changes have been reported in adolescents and young adults with risk factors for cardiovascular disease (CVD) (3, 4). The prevalence of CVD risk factors in childhood is associated with increased carotid artery intima-media thickness (IMT) in young adulthood (5–7), and this association is mainly from preadolescent age (8). Body growth and lean body mass (LBM) are major determinants of arterial wall layer thickness in preterm and term newborns (9) and during childhood (10, 11), and in adolescence, blood pressure (BP) influences the arterial wall through physiological remodeling (12, 13). Local body anthropometrics, including head circumference, are also related to child arterial wall layers and lumen diameters (LDs) (10). Maternal pre-pregnancy obesity has been linked to increased childhood body mass index (BMI) and BP reflected in carotid artery IMT (14).

Pre-eclamptic women develop an adverse CVD risk profile including elevated BP later in life (15, 16), and this is also observed in their offspring from childhood and young adulthood (17–19). However, a recent cohort study found no associations between hypertensive disorders of pregnancy (gestational hypertension or PE) and carotid IMT in children aged 10 years (20). To date, studies investigating the vascular structure and function of younger PE-exposed children are limited, likely reflecting the methodological challenges related to age and vessel size (21). Moreover, large cohorts on healthy children and adolescents have focused on the carotid artery only (11, 13). We have recently reported elevated BPs and increased regional pulsed wave velocity in preadolescent PE-exposed children aged 8–12 years (22). Our hypothesis was that elevated BPs impact the vascular health of preadolescent PE-exposed children. In this study, we aimed to assess the relations between BP, arterial structure, and local carotid artery stiffness in a preadolescent PE-exposed study population in relation to gestational and perinatal factors as well as child body anthropometrics and composition at follow-up. We then compared the results to an age-matched unexposed (non-PE) control group.

## 2. Materials and methods

### 2.1. Study design, sample, and setting

This study is part of the FINNCARE study (23), which is a follow-up of the Finnish Genetics of Pre-eclampsia Consortium (FINNPEC) multicenter study cohort (24). In 2008–2011, 1,450 nulli- or multiparous PE women and 1,065 non-PE women were prospectively recruited along with their partners and newborns. PE was defined as hypertension and proteinuria occurring after 20 weeks of gestation [systolic BP (SBP) ≥ 140 mmHg and/or diastolic BP (DBP)  ≥ 90 mmHg, urinary excretion of ≥0.3 g protein in a 24-h specimen or 0.3 g/L, or two ≥1+ readings on dipstick].

In the FINNCARE study (NCT04676295), PE-exposed and non-PE-exposed families from the FINNPEC cohort living in the Hospital District of Helsinki and Uusimaa were recalled and examined 8–12 years after delivery in a prospective cohort study setting to evaluate their CVD risk profile during preadolescence (Supplementary Figure S1). The exclusion criteria for all mothers included ongoing pregnancy or lactation, multiple pregnancy, and inability to communicate in Finnish. For non-PE-exposed families, the exclusion criteria also included PE, gestational hypertension or chronic hypertension, gestational diabetes, and/or diabetes during or following the index pregnancy. There were no statistical differences in major maternal gestational and child perinatal background characteristics between participating (*N* = 192) and non-participating (*N* = 118) PE dyads from the Hospital District of Helsinki and Uusimaa FINNPEC cohort precluding potential recruitment bias (results not shown). Data were collected from 182 PE-exposed and 85 non-PE-exposed children examined in a tertiary care setting at the Clinical Trial Unit located at Children's Hospital, Helsinki University Hospital, Finland, between June 2019 and June 2022. The FINNCARE study received ethical approval from the Ethics Committee of the Hospital District of Helsinki and Uusimaa in December 2018 (HUS/3347/2018), and participation was confirmed with a signed informed consent.

### 2.2. Arterial structure and local carotid stiffness

One skilled investigator (TS) obtained the vascular ultrasound images using the Vevo MD system (VisualSonics, Toronto, ON, Canada). The Vevo MD was equipped with electronic transducers (UHF48 and UHF70) corresponding to 30- and 50-MHz center frequencies, and the highest frequency able to image the far wall without compression was applied. Common carotid artery images were obtained 1 cm proximal to the carotid bulb, and femoral artery images were obtained at the inguinal fold. Both were examined bilaterally. Right radial artery images were assessed 1 cm proximal to the skin fold that separates the palma manus from the anterior antebrachium, and right brachial artery images were assessed 2–5 cm proximal to the cubital skin fold. The left radial artery was assessed, in substitution for the right radial artery, in six children because of right radial artery hypoplasia related to an arterial line during the neonatal stage. Bilateral radial arteries in one prematurely born child were hypoplastic, precluding radial artery analyses. Another investigator (MR) blinded to study subject characteristics analyzed the vascular images offline with electronic calipers using the VevoLAB software. IMT and LD during systole and diastole were measured for all blood vessels. Intima-media-adventitia thickness (IMAT) was measured in radial, brachial, and femoral arteries. Adventitia thickness (AT) was calculated as the mathematical difference of IMAT − IMT. We used the leading-edge technique, and all measurements, except systolic LD, were acquired in end-diastole (25). The mean of three caliper measurements was used in analyses. The right and left vessels provided similar results, and means are reported and used in analyses.

We calculated the common carotid artery β-stiffness index (CBSI, local carotid artery stiffness) (26), common carotid artery distensibility coefficient (CDC, local carotid artery distensibility) (26), and common carotid artery wall stress (CWS, local carotid artery wall stress) (27) using the following formulas:

$$CBSI\;=\;ln\left(\frac{SBP}{DBP}\right)/\left(\frac{CCALDS-CCALDD}{CCALDD}\right)$$

$$CDC\;=\;1000*\left(\frac{\left(\frac{CCALAS-CCALAD}{CCALAD}\right)}{SBP-DBP}\right)$$

CWS=(MAPxCCALDD)/(2xCCAIMT)

SBP, DBP, and MAP are office systolic BP, diastolic BP, and mean arterial pressure. Office BP was measured following a 1-h rest in the sitting position from the non-dominant arm with the Omron HBP-1300 and HBP-1320 devices. MAP was calculated as DBP + 1/3(SBP − DBP). CCALDS and CCALDD are common carotid artery lumen diameters in peak systole and end-diastole, respectively. CCALAS and CCALAD are common carotid artery lumen areas in systole and diastole, respectively. CCAIMT is common carotid artery IMT.

Child arterial wall layers and CBSI, CDC, and CWS variables are in the analyses used as early proxies of arterial health in our PE-exposed and non-PE-exposed children. Intra-variability coefficients of variation (CVs) were 1.7% (IMT) and 2.13% (LD) for the carotid artery, 5.1% (IMT) and 3.8% (IMAT) for the brachial artery, 3.2% (IMT) and 3.4% (IMAT) for the radial artery, and 3.0% (IMT) and 3.1% (IMAT) for the femoral artery. Inter-variability CVs were 5.2% (IMT) and 2.0% (LD) for the carotid artery, 9.3% (IMT) and 4.3% (IMAT) for the brachial artery, 4.1% (IMT) and 3.1% (IMAT) for the radial artery, and 3.9% (IMT) and 4.5% (IMAT) for the femoral artery.

### 2.3. Anthropometrics and body composition

Body height and weight were measured to the nearest 0.1 cm and 0.05 kg, respectively (Seca 285, Seca GmBH & Co, Hamburg, Germany). We measured waist, hip, thoracic, and head circumferences; arm and leg lengths; midpoint brachial, antebrachial, and calf circumferences; and thigh circumference (at the proximal one-third of the femur) to the nearest 0.1 cm using a tape measure. Bioelectrical impedance analysis (InBody 720, InBody Bldg, Korea) was performed to assess LBM, skeletal muscle mass, fat mass, and body fat percentage. The waist–hip ratio and BMI were calculated, as well as the body surface area (BSA) based on the Haycock formula. *Z*-scores for height, BMI and weight in relation to height, and weight in relation to age were generated based on a recent Finnish population dataset (28).

### 2.4. Ambulatory BP and pulse wave velocity

BP was measured at 30-min intervals during daytime and 1-h intervals at night for 24 h using an ambulatory BP oscillometric Schiller BR-102 plus device (29). The 24-h BP *z*-scores were calculated for height (30). Daytime and nighttime were corrected according to individual BP diaries, and 16 daytime and 18 nighttime registrations were excluded because less than 65% of the measurements were valid during the 24-h ambulatory recording (29). Office BP and ambulatory BP devices provided similar results when simultaneously compared in 36 children (results not shown).

Pulse wave velocity (PWV) was assessed at rest from the carotid–femoral (CF-PWV) and the carotid–radial (CR-PWV) regions with the mean of two measurements used in further analyses (Complior Analyse, Alam Medical, Saint-Quentin-Fallavier, France). The carotid–femoral distance was multiplied by 0.8. The Complior device automatically generated the central SBP, DBP, and pulse pressure (PP) based on the carotid waveform, and diastolic and mean office brachial BPs were used for calibration.

### 2.5. Questionnaires and index pregnancy data

Information on current household income (annual), child and parental background diseases, and parental smoking and alcohol intake was collected using standard questionnaires. We acquired maternal and perinatal data from the original FINNPEC database related to index pregnancy (24). Early-onset PE was defined as a PE diagnosis or delivery prior to 34^0/7^ gestational weeks and late-onset PE as a PE diagnosis or delivery at or after 34^0/7^ gestational weeks (31). Small for gestational age (SGA) was defined as birth weight below −2SD and prematurity as delivery before 37^0/7^ gestational weeks. We generated *z*-scores for birth height, birth weight, and birth head circumference based on a recent Finnish population dataset (32).

### 2.6. Data analysis

We present mean and standard deviation (SD), median and interquartile range (IQR), and count and percentage for normally distributed numerical data, non-normally distributed numerical data, and categorical data, respectively. Histograms, Kolmogorov–Smirnov and Shapiro–Wilk normality tests, Q–Q plots, and skewness were used to check for normality distribution. An independent-sample *t*-test or Mann–Whitney *U-*test was used to assess the differences between groups for continuous variables and Pearson *χ*^2^ or two-tailed Fisher's exact test to assess differences between groups for categorical variables.

We conducted univariate linear regression analyses to evaluate potential predictors for the vascular parameters of children. Potential predictors were organized into different domains in the tables: (1) child sex, (2) child age, (3) child anthropometrics, (4) child adiposity, (5) child ambulatory BP, and (6) child PWV. Only significant associations (*p* < 0.010) are reported because multiple tests in univariate regression analyses are prone to type 1 error.

Multiple linear regression models were then built to evaluate the influence of BP, PE, and other potential predictors on children's carotid, brachial, radial, and femoral arterial IMT, AT, and LD, as well as CBSI, CDC, and CWS. We included two models for peripheral artery variables to assess the influence of overall body size and local organ size. The unstandardized beta coefficients [and 95% confidence intervals (CIs)] were multiplied by 1,000 for all arterial wall layers (IMT and AT) in univariate and multiple linear regressions, showing the change in micrometers. We checked multiple linear regression models for normality, linearity, homoscedasticity, and independence. Multicollinearity was assessed with variance influence factor (VIF) and collinearity tolerance (CT), and VIF < 2.5 and CT > 0.3 were considered appropriate. Two-tailed tests were used in analyses, and a *p*-value of <0.05 was considered significant. SPSS v. 27 (IBM, New York, USA) was used for statistical analysis.

## 3. Results

### 3.1. Gestational, perinatal, and child follow-up characteristics

A total of 182 PE-exposed children and 85 non-PE-exposed children attended the follow-up visit, with four children being 8 years old; 24 children 9 years old; 61 children 10 years old; 81 children 11 years old; 75 children 12 years old; and 22 children 13 years old (mean age 11.4 years, results not shown). In total, 46 children were classified as early-onset PE based on diagnosis and 25 children based on the birth criteria (Table 1). PE-exposed children were more often premature and born SGA compared with non-PE-exposed children, which was accentuated in early-onset PE-exposed children.

**Table 1:** Child characteristics, anthropometrics, body composition, and blood pressure at follow-up.

	Non-PE	PE-exposed	Early-onset PE-exposed	Late-onset PE-exposed	*p*-value	Mean difference (95% CI)	*p*-value	Mean difference (95% CI)	*p*-value	Mean difference (95% CI)
			Diagnosis <34^0/7^ weeks	Diagnosis ≥34^0/7^ weeks	PE-exposed vs. non-PE		Early (dg) exposed vs. non-PE		Late (dg) exposed vs. non-PE	
**Characteristics and anthropometrics**	*N = 85*	*N = 182*	*N = 46*	*N = 136*	* *					
Premature, *n* (%)	4 (4.7)	60 (33.0)	42 (91.3)	18 (13.2)	**<0** **.** **001**	—	**<0** **.** **001**	—	**0** **.** **039**	—
SGA, *n* (%)	0 (0)	32 (17.6)	15 (32.6)	17 (12.5)	**<0** **.** **001**	—	**<0** **.** **001**	—	**<0** **.** **001**	—
Age (years)	11.2 (1.0)	11.6 (1.1)	11.6 (1.2)	11.6 (1.1)	**0** **.** **004**	0.4 (0.1 to 0.7)	**0** **.** **035**	0.4 (0–0.8)	**0** **.** **006**	0.4 (0.1–0.7)
Girls (*n*, %)	42 (49.4)	99 (54.4)	25 (54.3)	74 (54.4)	0.447	—	0.590	—	0.469	—
Body height (cm)	149.3 (8.4)	151.2 (10.1)	148.7 (9.1)	152.0 (10.3)	0.132	1.9 (−0.6 to 4.4)	0.713	−0.6 (−3.7 to 2.5)	**0** **.** **040**	2.7 (0.1 to 5.4)
Height *z*-score	0.15 (0.89)	0.05 (1.10)	−0.30 (1.18)	0.17 (1.06)	0.450	−0.10 (−0.37 to 0.17)	**0** **.** **015**	−0.45 (−0.81 to −0.09)	0.918	0.01 (−0.26 to 0.29)
Body weight (kg)	39.4 (10.4)^a^	40.3 (17.1)^a^	41.2 (18.5)^a^	40.1 (16.0)^a^	0.427	1.0 (−1.6 to 3.5)^a^	0.477	1.2 (−2.5 to 5.5)^a^	0.490	0.9 (−1.7 to 3.5)^a^
Weight *z*-score (height)	−0.13 (0.95)	−0.22 (1.10)	0.16 (0.97)	−0.35 (1.11)	0.502	−0.09 (−0.36 to 0.18)	0.102	0.29 (−0.06 to 0.63)	0.129	−0.22 (−0.51 to 0.06)
BMI (kg/m^2^)	17.8 (3.1)^a^	17.6 (4.1)^a^	18.6 (4.7)^a^	17.5 (4.0)^a^	0.993	−0.01 (−0.71 to 0.70)^a^	0.215	0.64 (−0.39 to 1.82)^a^	0.571	−0.19 (−0.93 to 0.50)^a^
BMI *z*-score	−0.02 (0.97)	−0.13 (1.08)	0.14 (1.05)	−0.22 (1.08)	0.443	−0.11 (−0.38 to 0.17)	0.381	0.16 (−0.20 to 0.52)	0.175	−0.20 (−0.48 to 0.09)
Body surface area (m^2^)^b^	1.30 (0.23)	1.33 (0.22)	1.33 (0.24)	1.33 (0.22)	0.228	0.03 (−0.02 to 0.08)	0.438	0.03 (−0.05 to 0.11)	0.255	0.03 (−0.02 to 0.08)
Waist circumference (cm)	63.0 (8.9)^a^	64.1 (11.0)^a^	67.0 (12.2)^a^	63.2 (10.5)^a^	0.331	1.0 (−1.0 to 3.0)^a^	0.109	2.7 (−0.5 to 5.9)^a^	0.605	0.5 (−1.5 to 2.5)^a^
Waist–hip ratio (no unit)	0.82 (0.05)	0.83 (0.06)	0.85 (0.07)	0.82 (0.06)	0.582	0 (−0.01 to 0.02)	**0** **.** **038**	0.02 (0 to 0.05)	0.737	0 (−0.02 to 0.01)
Hip circumference (cm)	77.95 (7.30)	79.82 (9.46)	80.45 (10.69)	79.61 (9.04)	0.079	1.87 (−0.22 to 3.96)	0.162	2.50 (−1.02 to 6.02)	0.137	1.66 (−0.53 to 3.85)
Head circumference (cm)	54.22 (1.76)	53.82 (1.70)	53.66 (1.94)	53.87 (1.61)	0.085	−0.40 (−0.86 to 0.06)	0.108	−0.56 (−1.24 to 0.12)	0.142	−0.35 (−0.82 to 0.12)
Arm length (cm)	49.00 (3.45)	49.34 (3.74)	48.19 (3.39)	49.72 (3.78)	0.491	0.34 (−0.63 to 1.30)	0.211	−0.81 (−2.08 to 0.46)	0.165	0.72 (−0.30 to 1.74)
Brachial circumference (cm)	22.63 (3.10)	22.97 (3.25)	23.50 (3.46)	22.79 (3.17)	0.439	0.33 (−0.51 to 1.18)	0.156	0.86 (−0.33 to 2.06)	0.725	0.16 (−0.72 to 1.03)
Antebrachial circumference (cm)	20.20 (2.19)	20.39 (2.10)	20.38 (2.16)	20.40 (2.09)	0.511	0.19 (−0.37 to 0.75)	0.672	0.17 (−0.63 to 0.98)	0.521	0.19 (−0.40 to 0.79)
Leg length (cm)	81.22 (6.38)	81.51 (6.98)	79.76 (7.35)	82.09 (6.78)	0.753	0.29 (−1.51 to 2.08)	0.250	−1.46 (−3.95 to 1.04)	0.354	0.87 (−0.98 to 2.71)
Thigh circumference (cm)	44.42 (5.50)	44.98 (5.89)	45.60 (6.24)	44.78 (5.78)	0.468	0.56 (−0.96 to 2.09)	0.275	1.18 (−0.95 to 3.32)	0.658	0.36 (−1.22 to 1.93)
Calf circumference (cm)	29.13 (2.80)	29.42 (3.52)	29.78 (3.50)	29.31 (3.53)	0.471	0.30 (0.41 to −0.51)	0.261	0.65 (−0.49 to 1.78)	0.684	0.18 (−0.68 to 1.04)
**Body composition**										
Skeletal muscle mass (kg)	17.2 (3.4)	17.9 (4.3)	17.4 (3.9)	18.0 (4.4)	0.162	0.7 (−0.3 to 1.6)	0.730	0.2 (−1.1 to 1.5)	0.115	0.8 (−0.2 to 1.9)
Lean mass bioimpedance (kg)	32.6 (5.7)	33.7 (7.2)	32.9 (6.6)	33.9 (7.4)	0.175	1.1 (−0.5 to 2.7)	0.737	0.4 (−1.8 to 2.6)	0.125	1.4 (−0.4 to 3.1)
Fat mass (kg)	6.3 (5.7)^a^	6.6 (7.1)^a^	7.5 (9.1)^a^	6.6 (5.6)^a^	0.914	0.1 (−1.0 to 1.1)^a^	0.291	0.9 (−0.7 to 2.9)^a^	0.734	−0.2 (−1.3 to 0.9)^a^
Body fat percentage (%)	19.0 (8.3)	18.9 (8.9)	21.2 (9.6)	18.1 (8.5)	0.920	−0.1 (−2.4 to 2.1)	0.170	2.2 (−1.0 to 5.4)	0.435	−0.9 (−3.2 to 1.4)
**24-h blood pressure**	*N = 63*	*N = 144*	*N = 35*	*N = 109*	* *					
SBP (mmHg)	119.6 (6.8)	122.5 (8.8)	125.1 (9.7)	121.7 (8.4)	**0** **.** **024**	2.9 (0.4 to 5.3)	**0** **.** **005**	5.5 (1.7 to 9.2)	0.107	2.0 (−0.4 to 4.5)
DBP (mmHg)	71.3 (5.4)	70.4 (5.8)	70.2 (6.3)	70.5 (5.6)	0.284	−0.9 (−2.6 to 0.8)	0.345	−1.2 (−3.5 to 1.3)	0.338	−0.8 (−2.6 to 0.9)
PP (mmHg)	48.4 (5.2)	52.1 (7.6)	55.1 (9.4)	51.1 (6.7)	**<0** **.** **001**	3.7 (1.9 to 5.4)	**<0** **.** **001**	6.7 (3.2 to 10.1)	**0** **.** **006**	2.7 (0.8 to 4.6)
HR (bpm)	81.6 (7.2)	80.9 (7.7)	81.7 (7.8)	80.6 (7.6)	0.534	−0.7 (1.1 to −3.0)	0.905	0.2 (−2.9 to 3.3)	0.400	−1.0 (−3.3 to 1.3)
SBP *z*-score (height)	1.26 (0.99)	1.60 (1.24)	2.07 (1.29)	1.45 (1.19)	0.058	0.34 (−0.01 to 0.69)	**<0** **.** **001**	0.81 (0.34 to 1.27)	0.295	0.19 (−0.16 to 0.54)
DBP *z*-score (height)	0.80 (0.99)	0.62 (1.05)	0.60 (1.09)	0.63 (1.04)	0.256	−0.18 (−0.49 to 0.13)	0.358	−0.20 (−0.63 to 0.23)	0.294	−0.17 (−0.49 to 0.15)
**Central blood pressure**	*N = 79*	*N = 174*	*N = 44*	*N = 130*						
Central SBP (mmHg)	103.0 (8.7)	109.7 (12.2)	112.8 (12.8)	108.6 (11.8)	**<0** **.** **001**	6.7 (3.7 to 9.7)	**<0** **.** **001**	9.8 (5.5 to 14.1)	**<0** **.** **001**	5.6 (2.8 to 8.4)
Central DBP (mmHg)	71.0 (6.1)	71.9 (6.0)	72.3 (6.4)	71.8 (5.9)	0.283	0.9 (−0.7 to 2.5)	0.269	1.3 (−1.0 to 3.6)	0.386	0.7 (−0.9 to 2.4)
Central PP (mmHg)	31.5 (10.0)^a^	36.6 (15.0)^a^	38.5 (16.0)^a^	36.3 (14.0)^a^	**<0** **.** **001**	5.0 (2.5 to 7.5)^a^	**<0** **.** **001**	7.0 (3.3 to 11.0)^a^	**0** **.** **001**	4.3 (1.7 to 7.0)^a^

dg, diagnosis; CI, confidence interval; SGA, small for gestational age (birth weight <−2SD); premature, birth <37^+0^ gestational weeks.

Data are presented as mean (SD) unless stated otherwise; significant *p*-values (<0.05) are in bold. Independent-sample *t*-test for normally distributed numerical data, Mann–Whitney *U*-test for non-normal distribution, and Pearson *χ*^2^ test or Fisher's exact test for categorical data.

^a^ Median (IQR), median difference (95% CI).

^b^ Calculated with Haycock's formula.

### 3.2. Vascular structure and function at the 8–12-year follow-up in the PE-exposed and non-PE-exposed groups

The LD, IMT, and AT values of carotid, brachial, radial, and femoral arteries were not statistically significantly different between PE-exposed (including early-onset and late-onset PE-exposed) and non-PE-exposed children (Table 2). CBSI, CDC, and CWS values were not different between PE-exposed (including PE-exposed subgroups) and non-PE-exposed children. Early-onset and late-onset PE-exposed children by delivery definition provided similar results for vascular structure and function (results not shown).

**Table 2:** Child vascular parameters at follow-up.

	Non-PE	PE-exposed	Early-onset exposed	Late-onset exposed	*p*-value		*p*-value		*p*-value	
			Diagnosis <34^0/7^ weeks	Diagnosis ≥34^0/7^ weeks	PE-exposed vs. non-PE	Mean difference (95% CI)	Early (dg) exposed vs. non-PE	Mean difference (95% CI)	Late (dg) exposed vs. non-PE	Mean difference (95% CI)
**Carotid artery (mm)**	*N = 85*	*N = 181*	*N = 46*	*N = 135*						
Lumen diameter (diastole)	4.84 (0.49)	4.74 (0.45)	5.67 (0.51)	5.65 (0.49)	0.128	−0.09 (−0.21 to 0.03)	0.231	−0.11 (−0.28 to 0.07)	0.171	−0.09 (−0.21 to 0.04)
Lumen diameter (systole)	5.71 (0.50)	5.65 (0.50)	4.73 (0.46)	4.75 (0.44)	0.413	−0.05 (−0.18 to 0.08)	0.683	−0.04 (−0.22 to 0.15)	0.390	−0.06 (−0.19 to 0.08)
Intima-media thickness	0.41 (0.05)	0.41 (0.06)	0.41 (0.06)	0.41 (0.05)	0.818	0 (−0.01 to 0.02)	0.958	0 (−0.02 to 0.02)	0.747	0 (−0.01 to 0.02)
**Brachial artery (mm)**	*N = 85*	*N = 180*	*N = 46*	*N = 134*						
Lumen diameter (diastole)	2.90 (0.30)	2.86 (0.37)	2.85 (0.37)	2.87 (0.38)	0.430	−0.04 (−0.13 to 0.05)	0.452	−0.05 (−0.16 to 0.07)	0.487	−0.03 (−0.13 to 0.06)
Intima-media thickness	0.11 (0.02)	0.11 (0.02)	0.11 (0.02)	0.11 (0.02)	0.456	0 (0 to 0.01)	0.560	0 (−0.01 to 0.01)	0.497	0 (0 to 0.01)
Intima-media-adventitia thickness	0.22 (0.04)	0.22 (0.03)	0.22 (0.03)	0.22 (0.03)	0.259	0.01 (0 to 0.01)	0.328	0.01 (−0.01 to 0.02)	0.331	0.01 (−0.01 to 0.01)
Adventitia thickness	0.11 (0.03)	0.11 (0.02)	0.11 (0.02)	0.11 (0.02)	0.336	0 (0 to 0.01)	0.396	0 (−0.01 to 0.01)	0.408	0 (0 to 0.01)
**Radial artery (mm)**	*N = 85*	*N = 179*	*N = 46*	*N = 133*						
Lumen diameter (diastole)	1.64 (0.21)	1.65 (0.25)	1.58 (0.24)	1.68 (0.24)	0.725	0.01 (−0.05 to 0.07)	0.144	−0.06 (−0.14 to 0.02)	0.272	0.04 (−0.03 to 0.10)
Intima-media thickness	0.12 (0.02)	0.13 (0.02)	0.13 (0.02)	0.13 (0.02)	0.117	0 (0 to 0.01)	0.431	0 (0 to 0.01)	0.093	0.01 (0 to 0.01)
Intima-media-adventitia thickness	0.19 (0.03)	0.20 (0.03)	0.19 (0.03)	0.20 (0.03)	0.088	0.01 (0 to 0.01)	0.325	0.01 (−0.01 to 0.02)	0.082	0.01 (0 to 0.02)
Adventitia thickness	0.07 (0.02)	0.07 (0.02)	0.07 (0.02)	0.07 (0.02)	0.280	0 (0 to 0.01)	0.437	0 (0 to 0.01)	0.301	0 (0 to 0.01)
**Femoral artery (mm)**	*N = 85*	*N = 181*	*N = 46*	*N = 135*						
Lumen diameter (diastole)	5.59 (0.67)	5.61 (0.65)	5.55 (0.66)	5.63 (0.65)	0.808	0.02 (−0.15 to 0.19)	0.784	−0.03 (−0.27 to 0.21)	0.664	0.04 (−0.14 to 0.22)
Intima-media thickness	0.21 (0.03)	0.21 (0.03)	0.21 (0.03)	0.22 (0.03)	0.266	0.01 (0 to 0.01)	0.761	0 (−0.01 to 0.01)	0.212	0.01 (0 to 0.02)
Intima-media-adventitia thickness	0.39 (0.06)	0.40 (0.06)	0.40 (0.05)	0.40 (0.06)	0.133	0.01 (0 to 0.03)	0.322	0.01 (−0.01 to 0.03)	0.147	0.01 (0 to 0.03)
Adventitia thickness	0.18 (0.04)	0.19 (0.04)	0.19 (0.04)	0.19 (0.04)	0.175	0.01 (0 to 0.02)	0.215	0.01 (−0.01 to 0.02)	0.238	0.01 (0 to 0.02)
**Arterial coefficients**	*N = 85*	*N = 181*	*N = 46*	*N = 135*						
Carotid beta stiffness index (no unit)	2.52 (0.68)	2.50 (0.53)	2.38 (0.42)	2.54 (0.55)	0.856	−0.02 (−0.18 to 0.15)	0.162	−0.14 (−0.33 to 0.06)	0.755	0.03 (−0.14 to 0.19)
Carotid distensibility coefficient (%/10 mmHg)	10.4 (2.8)	10.0 (2.6)	10.4 (2.1)	9.9 (2.7)	0.285	−0.37 (−1.05 to 0.31)	0.990	0.01 (−0.84 to 0.85)	0.189	−0.50 (−1.25 to 0.25)
Carotid wall stress (mmHg)	505 (84)	509 (89)	516 (105)	506 (84)	0.719	4.1 (−18.5 to 26.8)	0.510	11.1 (−22.1 to 44.3)	0.879	1.8 (−21.1 to 24.6)

Data are presented as mean (SD) unless stated otherwise; significant *p*-values (<0.05) are in bold. Independent-sample *t*-test for normally distributed numerical data and Mann–Whitney *U*-test for non-normal distribution.

### 3.3. Predictors of vascular structure and function among PE-exposed and non-PE-exposed children

Tables 3, 4 and Supplementary Tables S1, S2 show the univariate analyses exploring potential predictors of the arterial structure and function of all children. Brachial, femoral, and radial artery IMTs were associated with both ambulatory (24-h, daytime, and nighttime) and office central SBP and PP, while there were no associations with common carotid artery IMT and ambulatory BP or office central BP (Table 3). Radial artery IMT was also correlated with CF-PWV. Brachial and femoral artery IMTs were significantly related to general (height, weight, and thoracic circumference) and local (arm/leg lengths and circumferences) child anthropometric measures, with LBM and muscle mass showcasing the strongest associations. Radial artery IMT was associated with height, weight, BSA, head circumference, and leg and arm lengths and was strongest with LBM and skeletal muscle mass. Common carotid artery IMT was significantly associated with head circumference and height. Brachial and radial artery IMTs were higher among males. Brachial and femoral artery IMTs showed significant associations with adiposity measures BMI and BMI *z*-score, and brachial artery IMT with fat mass and waist–hip ratio. Radial artery IMT was only associated with fat mass percentage. However, no associations between adiposity and common carotid artery IMT were found. The relationship between peripheral artery AT and anthropometric measurements was weaker compared with IMT. However, significant associations were found between the femoral artery AT and BMI *z*-score, brachial artery AT and central SBP and PP, and radial artery AT and 24-h (including daytime and nighttime) SBP and central SBP and PP (Supplementary Table S1). Similar to IMTs, arterial lumen dimensions were strongly related to general and local child anthropometric measures, LBM and muscle mass, and BMI, waist–hip ratio, fat mass, and fat mass percentage (Supplementary Table S2). However, consistent relations between lumen dimensions and ambulatory BPs were not found.

**Table 3:** Univariate linear regression results for children's arterial intima-media thickness.

	Common carotid artery IMT				Brachial artery IMT				Radial artery IMT				Femoral artery IMT			
	*B* (95% CI)	Standβ	*R* ^2^	*p*	*B* (95% CI)	Standβ	*R* ^2^	*p*	*B* (95% CI)	Standβ	*R* ^2^	*p*	*B* (95% CI)	Standβ	*R* ^2^	*p*
Sex (0 = female, 1 = male)	12.03 (−0.70 to 24.77)	0.114	0.013	0.064	10.38 (5.85 to 14.91)	0.268	0.072	**<0.001**	8.69 (3.88 to 13.50)	0.215	0.046	**<0.001**	7.12 (−0.91 to 15.15)	0.107	0.011	0.082
Age (years)	−0.03 (−5.82 to 5.75)	−0.001	0.000	0.991	2.12 (0.01 to 4.22)	0.121	0.015	0.049	6.11 (4.02 to 8.20)	0.335	0.112	**<0.001**	6.03 (2.46 to 9.61)	0.201	0.040	**0.001**
Anthropometrics																
Body height (cm)	0.96 (0.30 to 1.62)	0.174	0.030	**0.004**	0.61 (0.38 to 0.85)	0.304	0.092	**<0.001**	0.80 (0.56 to 1.03)	0.378	0.143	**<0.001**	1.24 (0.85 to 1.63)	0.357	0.127	**<0.001**
Height *z*-score	11.62 (5.62 to 17.16)	0.229	0.052	**<0.001**	5.54 (3.38 to 7.69)	0.298	0.089	**<0.001**	3.81 (1.50 to 6.13)	0.197	0.039	**0.001**	9.31 (5.60 to 13.02)	0.291	0.085	**<0.001**
Body weight (kg)	0.45 (−0.12 to 1.02)	0.096	0.009	0.118	0.53 (0.34 to 0.73)	0.310	0.096	**<0.001**	0.38 (0.16 to 0.59)	0.209	0.044	**<0.001**	0.93 (0.59 to 1.28)	0.315	0.099	**<0.001**
Weight *z*-score (age)	7.72 (1.55 to 13.88)	0.150	0.022	0.014	5.53 (3.35 to 7.72)	0.294	0.086	**<0.001**	1.28 (−1.10 to 3.66)	0.065	0.004	0.292	9.27 (5.50 to 13.04)	0.286	0.082	**<0.001**
Body surface area (m^2^)	29.89 (−0.24 to 60.02)	0.119	0.014	0.052	29.32 (18.79 to 39.86)	0.320	0.102	**<0.001**	23.43 (12.15 to 34.70)	0.245	0.060	**<0.001**	53.52 (35.54 to 71.50)	0.339	0.115	**<0.001**
Lean body mass (kg)	1.15 (0.22 to 2.09)	0.148	0.022	0.016	0.99 (0.67 to 1.32)	0.352	0.124	**<0.001**	1.13 (0.79 to 1.46)	0.380	0.144	**<0.001**	1.90 (1.35 to 2.45)	0.384	0.148	**<0.001**
Skeletal muscle mass (kg)	1.98 (0.41 to 3.56)	0.151	0.023	0.014	1.70 (1.16 to 2.24)	0.357	0.128	**<0.001**	1.93 (1.36 to 2.49)	0.385	0.148	**<0.001**	3.19 (2.25 to 4.12)	0.383	0.146	**<0.001**
Head circumf (cm)	5.00 (1.25 to 8.76)	0.163	0.027	**0.009**	2.65 (1.29 to 4.00)	0.236	0.055	**<0.001**	1.97 (0.54 to 3.39)	0.170	0.029	**0.007**	4.63 (2.33 to 6.92)	0.242	0.059	**<0.001**
Thoracic circumf (cm)	0.92 (−0.04 to 1.87)	0.118	0.014	0.060	0.93 (0.60 to 1.26)	0.328	0.108	**<0.001**	0.38 (0.02 to 0.75)	0.131	0.017	0.038	1.18 (0.60 to 1.76)	0.244	0.060	**<0.001**
Hip circumf (cm)	0.37 (−0.35 to 1.10)	0.062	0.004	0.311	0.62 (0.36 to 0.87)	0.283	0.080	**<0.001**	0.31 (0.03 to 0.58)	0.136	0.018	0.028	1.15 (0.71 to 1.58)	0.306	0.093	**<0.001**
Brachial circumf (cm)	1.55 (−0.49 to 3.59)	0.093	0.009	0.137	1.43 (0.70 to 2.15)	0.236	0.056	**<0.001**	0.47 (−0.31 to 1.25)	0.075	0.006	0.233	2.54 (1.31 to 3.77)	0.247	0.061	**<0.001**
Antebrach circumf (cm)	3.99 (0.95 to 7.03)	0.160	0.026	0.010	2.19 (1.10 to 3.28)	0.241	0.058	**<0.001**	0.52 (−0.65 to 1.70)	0.055	0.003	0.380	3.99 (2.14 to 5.84)	0.258	0.067	**<0.001**
Arm length (cm)	2.60 (0.83 to 4.37)	0.179	0.032	**0.004**	1.59 (0.96 to 2.21)	0.299	0.090	**<0.001**	1.80 (1.15 to 2.45)	0.326	0.106	**<0.001**	2.75 (1.69 to 3.81)	0.305	0.093	**<0.001**
Thigh circumf (cm)	0.15 (−0.99 to 1.29)	0.016	0.000	0.798	0.57 (0.16 to 0.98)	0.170	0.029	**0.006**	0.21 (−0.22 to 0.65)	0.061	0.004	0.333	1.39 (0.71 to 2.07)	0.243	0.059	**<0.001**
Calf circumf (cm)	0.58 (−1.40 to 2.57)	0.036	0.001	0.563	1.54 (0.85 to 2.24)	0.264	0.070	**<0.001**	0.75 (0 to 1.50)	0.123	0.015	0.050	2.73 (1.55 to 3.91)	0.274	0.075	**<0.001**
Leg length (cm)	0.80 (−0.17 to 1.76)	0.102	0.010	0.104	0.65 (0.30 to 0.99)	0.226	0.051	**<0.001**	0.84 (0.48 to 1.19)	0.282	0.079	**<0.001**	1.38 (0.80 to 1.95)	0.284	0.081	**<0.001**
Adiposity																
Waist–hip ratio (no unit)	−40.58 (−154.41 to 73.25)	−0.043	0.002	0.483	54.81 (13.59 to 96.03)	0.159	0.025	**0.009**	5.19 (−38.51 to 48.89)	0.014	0.000	0.815	34.04 (−37.62 to 105.70)	0.057	0.003	0.350
BMI (kg/m^2^)	0.39 (−1.52 to 2.31)	0.025	0.001	0.687	1.35 (0.67 to 2.03)	0.233	0.054	**<0.001**	0.17 (−0.56 to 0.90)	0.028	0.001	0.648	2.06 (0.88 to 3.24)	0.206	0.043	**<0.001**
BMI *z*-score	3.38 (−2.73 to 9.49)	0.067	0.004	0.277	3.86 (1.67 to 6.06)	0.209	0.044	**<0.001**	−0.47 (−2.81 to 1.88)	−0.024	0.001	0.695	6.89 (3.12 to 10.65)	0.216	0.047	**<0.001**
Fat mass (kg)	−0.02 (−1.05 to 1.01)	−0.002	0.000	0.972	0.52 (0.15 to 0.89)	0.168	0.028	**0.006**	−0.13 (−0.52 to 0.27)	−0.039	0.002	0.529	0.82 (0.17 to 1.46)	0.152	0.023	0.013
Fat percentage (%)	−0.12 (−0.85 to 0.62)	−0.019	0.000	0.755	0.16 (−0.11 to 0.42)	0.072	0.005	0.243	−0.39 (−0.67 to −0.12)	−0.171	0.029	**0.006**	0.35 (−0.11 to 0.82)	0.092	0.008	0.136
Ambulatory BP (mmHg)																
24 h SBP	0.31 (−0.56 to 1.18)	0.049	0.002	0.480	0.45 (0.14 to 0.77)	0.193	0.037	**0.005**	0.45 (0.12 to 0.79)	0.183	0.033	**0.009**	0.90 (0.36 to 1.44)	0.222	0.049	**0.001**
24 h DBP	0.19 (−1.10 to 1.48)	0.020	0.000	0.773	−0.23 (−0.70 to 0.25)	−0.066	0.004	0.349	0.21 (−0.29 to 0.72)	0.058	0.003	0.406	0.08 (−0.75 to 0.90)	0.013	0.000	0.857
24 h PP	0.34 (−0.68 to 1.36)	0.046	0.002	0.514	0.73 (0.37 to 1.09)	0.267	0.071	**<0.001**	0.49 (0.09 to 0.88)	0.166	0.028	0.017	1.16 (0.53 to 1.80)	0.245	0.060	**<0.001**
Daytime SBP	0.23 (−0.59 to 1.04)	0.036	0.001	0.586	0.35 (0.06 to 0.64)	0.157	0.025	0.019	0.46 (0.14 to 0.77)	0.190	0.036	**0.004**	0.71 (0.20 to 1.21)	0.182	0.033	**0.006**
Daytime DBP	0.03 (−1.11 to 1.17)	0.004	0.000	0.957	−0.14 (−0.55 to 0.27)	−0.045	0.002	0.507	0.25 (−0.19 to 0.69)	0.074	0.006	0.268	0.08 (−0.64 to 0.79)	0.014	0.000	0.830
Daytime PP	0.37 (−0.65 to 1.39)	0.048	0.002	0.474	0.67 (0.31 to 1.02)	0.241	0.058	**<0.001**	0.51 (0.12 to 0.90)	0.169	0.029	**0.011**	1.03 (0.41 to 1.65)	0.213	0.045	**0.001**
Nighttime SBP	0.26 (−0.43 to 0.96)	0.051	0.003	0.452	0.30 (0.06 to 0.55)	0.160	0.026	0.017	0.28 (0.01 to 0.55)	0.139	0.019	0.039	0.56 (0.13 to 0.99)	0.170	0.029	0.011
Nighttime DBP	−0.02 (−1.00 to 0.95)	−0.003	0.000	0.964	−0.28 (−0.63 to 0.07)	−0.105	0.011	0.118	0.03 (−0.35 to 0.40)	0.009	0.000	0.889	−0.18 (−0.79 to 0.44)	−0.038	0.001	0.571
Nighttime PP	0.23 (−0.61 to 1.07)	0.036	0.001	0.591	0.69 (0.40 to 0.98)	0.303	0.092	**<0.001**	0.38 (0.06 to 0.70)	0.156	0.024	0.021	1.05 (0.54 to 1.56)	0.263	0.069	**<0.001**
PWV																
Carotid–femoral PWV (m/s)	−2.68 (−11.28 to 5.92)	−0.039	0.002	0.540	2.79 (−0.38 to 5.95)	0.110	0.012	0.084	7.55 (4.37 to 10.73)	0.284	0.081	**<0.001**	6.42 (1.15 to 11.70)	0.150	0.023	0.017
Carotid–radial PWV (m/s)	2.03 (−3.63 to 7.69)	0.045	0.002	0.481	1.41 (−0.68 to 3.50)	0.084	0.007	0.184	3.02 (0.87 to 5.17)	0.173	0.030	**0.006**	1.13 (−2.37 to 4.64)	0.040	0.002	0.525
Central SBP (mmHg)	0.35 (−0.21 to 0.92)	0.077	0.006	0.222	0.31 (0.11 to 0.52)	0.187	0.035	**0.003**	0.45 (0.24 to 0.66)	0.256	0.066	**<0.001**	0.50 (0.15 to 0.85)	0.177	0.031	**0.005**
Central DBP (mmHg)	0.34 (−0.75 to 1.42)	0.039	0.001	0.543	0.10 (−0.30 to 0.50)	0.031	0.001	0.620	0.34 (−0.07 to 0.76)	0.103	0.011	0.105	0.31 (−0.36 to 0.98)	0.057	0.003	0.364
Central PP (mmHg)	0.28 (−0.31 to 0.87)	0.060	0.004	0.345	0.31 (0.10 to 0.52)	0.177	0.031	**0.005**	0.38 (0.16 to 0.61)	0.210	0.044	**<0.001**	0.45 (0.09 to 0.81)	0.152	0.023	0.015

*B*, unstandardized beta; Standβ, standardized beta; 95% CI, 95% confidence interval; *R*^2^, non-adjusted *R* square; circumf, circumference.

The unstandardized coefficients (and 95% CIs) have been multiplied by 1,000, showing the change in micrometers.

Significant *p*-values (<0.01) are in bold.

**Table 4:** Univariate linear regression results for children's local carotid artery stiffness, distensibility, and wall stress.

	Local carotid artery stiffness				Local carotid artery distensibility				Local carotid artery wall stress			
	*B* (95% CI)	Standβ	*R* ^2^	*p*	*B* (95% CI)	Standβ	*R* ^2^	*p*	*B* (95% CI)	Standβ	*R* ^2^	*p*
Sex (0 = female, 1 = male)	0.03 (−0.11 to 0.17)	0.027	0.001	0.661	−0.07 (−0.71 to 0.57)	−0.013	0.000	0.831	5.12 (−16.05 to 26.29)	0.029	0.001	0.635
Age (years)	0.06 (0 to 0.13)	0.120	0.014	0.051	−0.34 (−0.63 to −0.05)	−0.143	0.020	0.020	0.63 (−8.93 to 10.19)	0.008	0.000	0.897
Anthropometrics												
Body height (cm)	0 (0 to 0.01)	0.058	0.003	0.348	−0.03 (−0.06 to 0.01)	−0.098	0.010	0.110	−0.04 (−1.15 to 1.06)	−0.005	0.000	0.939
Height *z*-score	−0.02 (−0.09 to 0.05)	−0.032	0.001	0.603	0 (−0.31 to 0.30)	−0.001	0.000	0.985	−0.80 (−10.98 to 9.38)	−0.010	0.000	0.877
Body weight (kg)	0.01 (0 to 0.01)	0.098	0.010	0.113	−0.04 (−0.07 to −0.02)	−0.189	0.036	**0** **.** **002**	1.17 (0.24 to 2.10)	0.150	0.023	**0** **.** **014**
Weight *z*-score (age)	0.04 (−0.03 to 0.11)	0.070	0.005	0.259	−0.37 (−0.68 to −0.06)	−0.143	0.021	0.019	11.41 (1.20 to 21.63)	0.134	0.018	0.029
Body surface area (m^2^)	0.27 (−0.07 to 0.61)	0.098	0.010	0.114	−2.25 (−3.73 to −0.76)	−0.180	0.032	**0** **.** **003**	50.86 (1.11 to 100.62)	0.123	0.015	0.045
Lean body mass (kg)	0.01 (−0.01 to 0.02)	0.055	0.003	0.372	−0.05 (−0.10 to 0)	−0.126	0.016	0.041	1.03 (−0.53 to 2.59)	0.080	0.006	0.194
Skeletal muscle mass (kg)	0.01 (−0.01 to 0.03)	0.053	0.003	0.393	−0.08 (−0.16 to 0)	−0.124	0.015	0.043	1.68 (−0.96 to 4.31)	0.077	0.006	0.212
Head circumference (cm)	0.03 (−0.02 to 0.07)	0.080	0.006	0.206	−0.24 (−0.43 to −0.06)	−0.163	0.027	**0** **.** **009**	8.66 (2.51 to 14.80)	0.172	0.030	**0** **.** **006**
Adiposity												
Waist circumference (cm)	0.01 (0 to 0.02)	0.137	0.019	0.027	−0.07 (−0.10 to −0.04)	−0.252	0.064	**<0** **.** **001**	1.93 (0.89 to 2.97)	0.219	0.048	**<0** **.** **001**
Waist–hip ratio (no unit)	1.48 (0.23 to 2.73)	0.143	0.021	0.020	−11.17 (−16.69 to −5.66)	−0.239	0.057	**<0** **.** **001**	301 (116 to 485)	0.193	0.037	**0** **.** **002**
BMI (kg/m^2^)	0.02 (0 to 0.04)	0.120	0.014	0.051	−0.17 (−0.26 to −0.08)	−0.217	0.047	**<0** **.** **001**	5.52 (2.43 to 8.62)	0.211	0.045	**<0** **.** **001**
BMI z-score	0.06 (−0.01 to 0.12)	0.100	0.010	0.105	−0.43 (−0.73 to −0.13)	−0.172	0.030	**0** **.** **005**	14.55 (4.59 to 24.52)	0.174	0.030	**0** **.** **004**
Fat mass (kg)	0.01 (0 to 0.02)	0.103	0.011	0.096	−0.08 (−0.13 to −0.03)	−0.197	0.039	**0** **.** **001**	2.77 (1.10 to 4.44)	0.197	0.039	**0** **.** **001**
Fat (%)	0.01 (0 to 0.02)	0.110	0.012	0.076	−0.05 (−0.09 to −0.02)	−0.177	0.031	**0** **.** **004**	1.65 (0.46 to 2.85)	0.165	0.027	**0** **.** **007**
PWV												
Carotid–femoral PWV (m/s)	−0.07 (−0.16 to 0.03)	−0.086	0.007	0.176	−0.04 (−0.47 to 0.38)	−0.013	0.000	0.842	19.35 (5.47 to 33.22)	0.171	0.029	**0** **.** **006**
Carotid–radial PWV (m/s)	−0.06 (−0.12 to 0.01)	−0.112	0.013	0.077	0.11 (−0.17 to 0.39)	0.049	0.002	0.440	6.43 (−2.82 to 15.68)	0.087	0.008	0.172

*B*, unstandardized beta; Standβ, standardized beta; 95% CI, 95% confidence interval; *R*^2^, non-adjusted *R* square.

Significant *p*-values (<0.01) are in bold.

CDC and CWS parameters were associated with adiposity parameters, including waist circumference, waist–hip ratio, BMI, BMI *z*-score, fat mass, and fat mass percentage (Table 4). CDC was also associated with anthropometrics, including body weight, BSA and head circumference, and CWS with head circumference only. CDC further showcased borderline significance with child age. CWS was the only local carotid artery parameter associated with CF-PWV. CBSI was only borderline associated with waist circumference and waist–hip ratio, while no consistent associations with other child anthropometrics were found. However, when data were analyzed in children exposed to PE only, CBSI was associated with BMI, fat mass, and fat mass percentage (results not shown). Parameters for local carotid artery stiffness were not associated with sex.

Table 5 and Supplementary Table S3 show multiple linear regression models assessing the influence of multiple predictors on arterial structure and local carotid artery function in PE-exposed and non-PE-exposed children combined. All peripheral arteries’ IMTs, including brachial, radial, and femoral, were independently predicted by child LBM and 24-h SBP at the follow-up (Table 5). LBM displayed the highest standardized coefficients in all peripheral artery IMT models (brachial artery IMT model's adjusted *R*^2^ 0.217, radial artery IMT model's adjusted *R*^2^ 0.208, and femoral artery IMT model's adjusted *R*^2^ 0.214). The local organ size performed similarly to LBM, or slightly less, and remained significantly related to the IMTs of all peripheral arteries. Furthermore, brachial and radial artery IMTs were higher in males, and radial artery IMT was related to child age at follow-up. PE was non-significant in all models. The femoral artery IMT model was stronger using 24-h SBP compared with office central SBP (LBM model: adjusted *R*^2^ 0.214 vs. 0.127; local organ size model: *R*^2^ 0.172 vs. 0.070). Like IMTs, peripheral artery LDs were independently predicted by sex, LBM, and local organ size (Supplementary Table S3; brachial artery model adjusted *R*^2^ 0.431, radial artery model adjusted *R*^2^ 0.211, and femoral artery model adjusted *R*^2^ 0.394). PE was not related to LDs in any models. Central SBP was not a significant predictor of vascular wall layer thickness or LD (results not shown).

**Table 5:** Multiple linear regression models for children's arterial intima-media thickness and carotid artery stiffness, distensibility, and wall stress.

			Unstandardized				
Vascular dimension		Predictor	β (95% CI)	Standardized β	*p*-value	Adjusted *R*^2^	Model *p*-value
Brachial artery							
* *Intima-media thickness	Model 1	Constant	29.12 (−5.51 to 63.75)			0.217	**<0** **.** **001**
		Pre-eclampsia-exposed (0 = no, 1 = yes)	0.19 (−4.96 to 5.34)	0.005	0.942		
		Child sex (0 = female, 1 = male)	7.79 (3.09 to 12.50)	0.204	**0** **.** **001**		
		Child lean body mass at follow-up (kg)	0.99 (0.65 to 1.32)	0.368	**<0** **.** **001**		
		Child 24-h SBP at follow-up (mmHg)	0.36 (0.08 to 0.65)	0.159	**0** **.** **013**		
	Model 2	Constant	−26.38 (−74.90 to 22.14)			0.181	**<0** **.** **001**
		Pre-eclampsia-exposed (0 = no, 1 = yes)	1.29 (4.22 to 6.80)	0.030	0.644		
		Child sex (0 = female, 1 = male)	8.78 (3.74 to 13.81)	0.223	**<0** **.** **001**		
		Child arm length at follow-up (cm)	1.64 (0.98 to 2.31)	0.314	**<0** **.** **001**		
		Child 24-h SBP at follow-up (mmHg)	0.42 (0.12 to 0.72)	0.179	**0** **.** **006**		
Radial artery							
* *Intima-media thickness	Model 1	Constant	12.89 (−35.40 to 61.19)			0.208	**<0** **.** **001**
		Pre-eclampsia-exposed (0 = no, 1 = yes)	1.43 (−4.22 to 7.09)	0.032	0.617		
		Child age at follow-up (years)	3.36 (0.24 to 6.48)	0.177	**0** **.** **035**		
		Child sex (0 = female, 1 = male)	8.11 (2.90 to 13.33)	0.196	**0** **.** **002**		
		Child lean body mass at follow-up (kg)	0.72 (0.24 to 1.20)	0.249	**0** **.** **003**		
		Child 24-h SBP at follow-up (mmHg)	0.37 (0.06 to 0.68)	0.149	**0** **.** **021**		
	Model 2	Constant	−34.83 (−87.58 to 17.91)			0.199	**<0** **.** **001**
		Pre-eclampsia-exposed (0 = no, 1 = yes)	2.12 (−3.74 to 7.98)	0.047	0.476		
		Child age at follow-up (years)	4.01 (0.94 to 7.07)	0.199	**0** **.** **011**		
		Child sex (0 = female, 1 = male)	9.27 (3.95 to 14.60)	0.223	**<0** **.** **001**		
		Child arm length at follow-up (cm)	1.20 (0.37 to 2.02)	0.217	**0** **.** **005**		
		Child 24-h SBP at follow-up (mmHg)	0.41 (0.10 to 0.72)	0.166	**0** **.** **011**		
Femoral artery							
Intima-media thickness	Model 1	Constant	64.55 (3.09 to 126.01)			0.214	**<0** **.** **001**
		Pre-eclampsia-exposed (0 = no, 1 = yes)	−2.40 (−11.49 to 6.69)	−0.033	0.604		
		Child lean body mass at follow-up (kg)	2.02 (1.43 to 2.61)	0.424	**<0** **.** **001**		
		Child 24-h SBP at follow-up (mmHg)	0.69 (0.18 to 1.19)	0.169	**0** **.** **008**		
	Model 2	Constant	16.41 (−52.20 to 85.02)			0.172	**<0** **.** **001**
		Pre-eclampsia-exposed (0 = no, 1 = yes)	−3.64 (−12.98 to 5.70)	−0.050	0.443		
		Child calf circumference at follow-up (cm)	3.59 (2.33 to 4.86)	0.365	**<0** **.** **001**		
		Child 24-h SBP at follow-up (mmHg)	0.78 (0.27 to 1.29)	0.198	**0** **.** **003**		
CBSI	Model 1	Constant	0.61 (−0.63 to 1.85)			0.024	**0** **.** **026**
		Pre-eclampsia-exposed (0 = no, 1 = yes)	−0.05 (−0.20 to 0.10)	−0.039	0.530		
		Child age at follow-up (years)	0.06 (0 to 0.13)	0.122	**0** **.** **051**		
		Child waist–hip ratio at follow-up	1.45 (0.20 to 2.70)	0.140	**0** **.** **023**		
					
CDC	Model 1	Constant	22.81 (17.34 to 28.27)			0.066	**<0** **.** **001**
		Pre-eclampsia-exposed (0 = no, 1 = yes)	−0.20 (−0.87 to 0.47)	−0.036	0.555		
		Child age at follow-up (years)	−0.31 (−0.59 to −0.02)	−0.128	**0** **.** **035**		
		Child waist–hip ratio at follow-up	−10.91 (−16.39 to −5.43)	−0.233	**<0** **.** **001**		
CWS	Model 1	Constant	−99.19 (−441.85 to 243.47)			0.047	**0** **.** **002**
		Pre-eclampsia-exposed (0 = no, 1 = yes)	9.24 (−13.58 to 32.05)	0.049	0.426		
		Child head circumference at follow-up (cm)	7.19 (0.92 to 13.45)	0.143	**0** **.** **025**		
		Child waist–hip ratio at follow-up	254.15 (61.36 to 446.93)	0.163	**0** **.** **010**		

The unstandardized coefficients (and 95% CIs) of intima-media thickness have been multiplied by 1,000, showing the change in micrometers. Significant results are in bold (*p*-value <0.05).

CBSI, CDC, and CWS were all independently predicted by the waist–hip ratio at follow-up (Table 5, model's adjusted *R*^2^ 0.024, 0.066, and 0.047, respectively). CDC and CWS were also independently predicted by body fat percentage with model's adjusted *R*^2^ 0.036 and 0.043, respectively (results not shown). CDC was independently predicted by child age at follow-up, and for CBSI, age reached borderline significance as a predictor. CWS was predicted by the child head circumference.

Multiple significant associations were observed between carotid and peripheral artery LDs and IMTs and different birth anthropometrics (height, weight, and head circumference), but birth anthropometrics did not remain significant when adjusting for child anthropometrics at follow-up (results not shown). Similarly, we found no independent associations between LDs and IMTs at follow-up and maternal pre-pregnancy BMI, age at delivery, parity, maternal gestational BPs, child prematurity, or SGA (results not shown). Similarly, no relations were seen for CBSI, CDC, and CWS (results not shown).

## 4. Discussion

In this study, we report no differences in the vascular structure or CBSI, CDC, and CWS in 8- to 12-year-old PE-exposed children compared with non-PE-exposed children with lower SBPs and similar sex distribution, age, body size, and body adiposity composition at follow-up. Nevertheless, an independent positive association between SBP (and PP) and peripheral artery, but not with carotid artery wall thickness, is shown in PE-exposed and non-PE-exposed children, indicating BP-related arterial remodeling during prepubertal age. In addition, the waist–hip ratio was marginally higher in the early-onset PE-exposed group, and the waist–hip ratio, as well as other adiposity measures, showed independent positive associations with PE-exposed and non-PE-exposed children's CBSI, CDC, and CWS. Taken together, these surrogate CVD marker results indicate early, although mild, progression of CVD in young PE-exposed children, adding to previous studies showing increased arterial IMT in young adulthood being linked with childhood CVD risk factors (5–8).

Although PE-exposed children in the present study showed increased SBP (and PP), no differences between PE-exposed and non-PE-exposed arterial wall layer thickness were found. This finding is similar to recent small sample size PE studies of similar child age groups that show no PE-related difference in carotid IMT and distensibility (20, 33). However, reports are showing BP-related increased carotid IMT later in life in young adults born from preterm hypertensive pregnancies (either gestational hypertension or PE) compared with preterm normotensive pregnancies (34). PE is associated with the early development of CVD in offspring during adulthood (35, 36).

In our analyses of arterial IMTs in children, we assess the relation between BP and arterial media thickness since more than 95% of the IMT complex in prepubertal children correspond to the muscular medial layer thickness (25). Our interpretation shows that the positive independent arterial layer associations with SBP are then due to remodeling of the medial layer in response to BP-related wall stress and do not reflect changes in intima layer thickness. The lower augmentation of the arterial waveform in the more central carotid artery compared with the peripheral artery may explain the lack of BP-related medial thickening in the carotid artery compared with our peripheral artery results (12). Furthermore, our results show that the BP-related arterial wall changes are not attributed to maternal, gestational, and prematurity factors or child adiposity at follow-up. Results of the regression analyses suggest LBM to be a major predictor of arterial wall thickness in preadolescent children, while the contribution of SBP is roughly half of this, like the contribution of the male sex. This is consistent with previous reports in younger and older healthy children and adolescents (10, 12, 13). The present study adds to the literature by showing LBM to be a stronger predictor of peripheral artery wall layer thickness compared with local limb organ size. However, for the children's carotid artery wall and lumen dimensions, head circumference seems to be an even stronger predictor than LBM or BSA and is likely explained by the different head and overall body growth trajectories with head circumference growth trajectory being non-linear (37). Head circumference has been previously shown to be associated with carotid artery LD in univariate regression analyses in healthy children (10), and the present study further adds to the literature by showing head circumference as an independent predictor in multiple linear regression models for carotid artery dimensions. Our results are also consistent with previous studies that show body size and composition-independent associations between severity of primary hypertension and carotid IMT (38). In our recent publication on the same study sample of children, we have not been able to show any differences in blood fasting glucose, insulin, lipids, and high-sensitivity C-reactive protein (hs-CRP) between PE-exposed and non-PE-exposed children. Also, no statistically significant relations between blood fasting glucose, insulin, lipids, hs-CRP levels, and arterial layer thickness were observed among PE-exposed and non-PE-exposed children (results not shown) (22). To date, studies investigating the arterial health of PE-exposed children are limited, and the present study then adds to the literature showing early BP-related changes in arterial wall thickness in prepubertal children.

The present study reports independent associations between adiposity measures and CBSI, CDC, and CWS at follow-up. Previous studies have also shown different adiposity measures associated with carotid distensibility and wall stress in healthy children and adolescents (11, 13). We have recently reported office SBP-related elevations in carotid–femoral PWVs in the same PE-exposed child study population (22). The significant associations between PWV and peripheral artery IMTs are then likely mediated by SBP. Taken together, prepubertal children show increased arterial stiffness in the carotid and other regional arteries.

Our novel ultra-high-frequency ultrasound technique also allowed the assessment of peripheral adventitial thickness. However, only weak non-independent associations with body anthropometric, LBM, and SBP variables were found. Previous reports in healthy non-obese children using an identical ultrasound methodology show arterial wall growth mainly attributed to the medial layer and not the adventitial layer (12). The small adventitial thickness measurement is also inevitably associated with a technical variance that limits the assessment of biological variance and differences.

The major strengths of the study include the prospective study design, large sample size, comprehensive assessment of BP, and use of novel ultrasound histology-validated methodology to assess arterial wall layer thickness in multiple arterial sites, accounting for body anthropometrics and composition at follow-up as well as maternal, gestational, and perinatal factors. Limitations include a lack of data regarding postnatal child growth.

In conclusion, although we found no difference in arterial structure and local carotid artery stiffness between prepubertal PE-exposed and non-PE-exposed children, higher SBP and PP in PE-exposed children reflect increased peripheral artery IMT, suggesting mainly increased muscular media thickness, but not in the carotid artery. CBSI, CDC, and CWS are independently related to adiposity, and carotid artery dimensions are strongly predicted by the head circumference of a child. Overall, PE-exposed children display unfavorable BP and adiposity-related associations in vascular health consistent with early progression of CVD.

## Data Availability

The raw data supporting the conclusions of this article will be made available by the authors, without undue reservation.
