# Supplementary material for: Determinants of vascular structure and function in at-risk children born to mothers managed for pre-eclampsia (FINNCARE study)
Source: Front Cardiovasc Med. 2023 Oct 4;10:1264921. doi: 10.3389/fcvm.2023.1264921 (PMC10582712; doi:10.3389/fcvm.2023.1264921)
Supplement: Supplementary file 1 [file Datasheet1.pdf]

Supplementary Material

Determinants of vascular structure and function in at-risk children born to mothers managed for pre-eclampsia (FINNCARE-study)

Michelle Renlund*, Tiina Jääskeläinen, Anni Kivelä, Seppo Heinonen, Hannele Laivuori, and Taisto Sarkola

*** Correspondence:** Michelle Renlund, MD: michelle.renlund@helsinki.fi

1 Supplementary Figures and Tables

Supplementary Table 1. Univariate linear regression results for children's arterial adventitia thickness

		Brachial artery AT				Radial artery AT				Femoral artery AT			
		B (95% CI)	Standardized β	R^2	*p*	B (95% CI)	Standardized β	R^2	*p*	B (95% CI)	Standardized β	R^2	*p*
Sex (0 = female, 1 = male)	**Sex**	0.04 (-6.04-6.11)	0.001	0.000	0.991	0.21 (-4.18-4.61)	0.006	0.000	0.924	1.41 (-8.17-10.99)	0.018	0.000	0.772
Age (years)	**Age**	0.76 (-1.98-3.50)	0.034	0.001	0.585	2.31 (0.35-4.27)	0.142	0.020	0.021	1.09 (-3.23-5.41)	0.031	0.001	0.620
Body height (cm)		0.36 (0.05-0.68)	0.140	0.020	0.023	0.27 (0.04-0.50)	0.144	0.021	0.019	0.51 (0.02-1.01)	0.124	0.015	0.043
Height z-score		3.84 (0.96-6.72)	0.160	0.026	**0.009**	1.13 (-0.97-3.23)	0.065	0.004	0.291	5.65 (1.10-10.20)	0.149	0.022	0.015
Body weight (kg)		0.27 (0-0.53)	0.120	0.014	0.052	0.12 (-0.08-0.31)	0.073	0.005	0.239	0.51 (0.09-0.93)	0.146	0.021	0.017
Weight z-score (height)		0.86 (-2.03-3.75)	0.036	0.001	0.558	-0.47 (-2.56-1.62)	-0.027	0.001	0.658	5.59 (1.08-10.10)	0.149	0.022	0.015
Weight z-score (age)		3.01 (0.09-5.94)	0.124	0.015	0.044	0.39 (-1.74-2.52)	0.022	0.000	0.718	7.53 (2.96-12.10)	0.196	0.038	**0.001**
Body surface area (m^2)		15.00 (0.75-29.24)	0.127	0.016	0.039	7.68 (-2.66-18.02)	0.090	0.008	0.145	28.72 (6.31-51.13)	0.153	0.024	0.012
Lean body mass (kg)		0.52 (0.08-0.97)	0.141	0.020	0.022	0.36 (0.04-0.69)	0.136	0.018	0.027	0.79 (0.09-1.50)	0.136	0.018	0.027
Skeletal muscle mass (kg)		0.87 (0.12-1.62)	0.139	0.019	0.024	0.60 (0.06-1.14)	0.133	0.018	0.031	1.31 (0.12-2.50)	0.133	0.018	0.031
Head circumference (cm)	**Anthropometrics**	1.94 (0.19-3.70)	0.136	0.019	0.030	0.44 (-0.86-1.74)	0.042	0.002	0.502	3.60 (0.85-6.36)	0.160	0.026	0.011
Thoracic circumference (cm)		0.50 (0.06-0.94)	0.139	0.019	0.026	0.16 (-0.17-0.49)	0.060	0.004	0.344	0.99 (0.30-1.69)	0.175	0.030	**0.005**
Hip circumference (cm)		0.28 (-0.06-0.62)	0.099	0.010	0.108	0.08 (-0.17-0.33)	0.040	0.002	0.521	0.87 (0.34-1.40)	0.195	0.038	**0.001**
Brachial circumference (cm)		0.92 (-0.02-1.85)	0.120	0.014	0.056	0.18 (-0.51-0.88)	0.033	0.001	0.603	1.67 (0.19-3.15)	0.138	0.019	0.027
Antebrachial circumference (cm)		1.93 (0.52-3.33)	0.167	0.028	**0.007**	0.27 (-0.78-1.31)	0.032	0.001	0.614	2.86 (0.64-5.09)	0.157	0.025	0.012
Arm length (cm)		1.01 (0.19-1.83)	0.151	0.023	0.016	0.68 (0.08-1.29)	0.138	0.019	0.028	1.80 (0.51-3.10)	0.169	0.029	**0.007**
Thigh circumference (cm)		0.48 (-0.04-1.00)	0.113	0.013	0.070	0.15 (-0.23-0.54)	0.049	0.002	0.433	0.90 (0.08-1.73)	0.134	0.018	0.032
Calf circumference (cm)		0.92 (0.01-1.83)	0.125	0.016	0.047	0.49 (-0.18-1.16)	0.090	0.008	0.152	2.16 (0.74-3.59)	0.184	0.034	**0.003**
Leg length (cm)		0.26 (-0.19-0.71)	0.072	0.005	0.250	0.30 (-0.03-0.62)	0.112	0.013	0.075	0.55 (-0.16-1.25)	0.096	0.009	0.127
Waist-hip ratio (no unit)		6.45 (-47.45-60.35)	0.015	0.000	0.814	-1.40 (-40.44-37.63)	-0.004	0.000	0.944	34.58 (-50.46-119.61)	0.049	0.002	0.424
BMI (kg/m^2)		0.51 (-0.40-1.41)	0.068	0.005	0.272	0.04 (-0.62-0.69)	0.007	0.000	0.908	1.57 (0.15-2.99)	0.132	0.018	0.031
BMI z-score	**Adiposity**	1.71 (-1.18-4.60)	0.072	0.005	0.245	-0.06 (-2.16-2.04)	-0.003	0.000	0.955	6.75 (2.25-11.26)	0.179	0.032	**0.003**
Fat mass (kg)		0.23 (-0.26-0.72)	0.056	0.003	0.362	-0.06 (-0.42-0.29)	-0.021	0.000	0.735	0.75 (-0.02-1.52)	0.118	0.014	0.055
Fat percentage (%)		0.04 (-0.31-0.39)	0.014	0.000	0.823	-0.13 (-0.38-0.12)	-0.063	0.004	0.309	0.66 (0.11-1.21)	0.145	0.021	0.018
24 hours SBP (mmHg)		0.22 (-0.17-0.61)	0.077	0.006	0.271	0.41 (0.12-0.69)	0.193	0.037	**0.005**	0.40 (-0.22-1.02)	0.089	0.008	0.202
24 hours DBP (mmHg)		0.05 (-0.53-0.64)	0.013	0.000	0.855	0.29 (-0.14-0.72)	0.092	0.009	0.187	0.25 (-0.67-1.17)	0.037	0.001	0.592
24 hours PP (mmHg)		0.30 (-0.16-0.76)	0.090	0.008	0.196	0.38 (0.04-0.72)	0.154	0.024	0.027	0.41 (-0.32-1.13)	0.077	0.006	0.269
Daytime SBP (mmHg)		0.30 (-0.09-0.68)	0.100	0.010	0.134	0.41 (0.14-0.68)	0.196	0.039	**0.003**	0.32 (-0.26-0.90)	0.073	0.005	0.278
Daytime DBP (mmHg)	**Ambulatory BP**	0.17 (-0.37-0.71)	0.042	0.002	0.529	0.42 (0.04-0.80)	0.146	0.021	0.029	0.26 (-0.55-1.06)	0.041	0.002	0.536
Daytime PP (mmHg)		0.30 (-0.19-0.78)	0.081	0.006	0.230	0.30 (-0.04-0.64)	0.117	0.014	0.080	0.30 (-0.43-1.02)	0.054	0.003	0.417
Nighttime SBP (mmHg)		0.08 (-0.23-0.38)	0.034	0.001	0.612	0.34 (0.11-0.56)	0.196	0.038	**0.003**	0.34 (-0.15-0.82)	0.091	0.008	0.177
Nighttime DBP (mmHg)		-0.30 (-0.74-0.13)	-0.094	0.009	0.164	0.19 (-0.13-0.51)	0.079	0.006	0.241	0.23 (-0.46-0.92)	0.045	0.002	0.508
Nighttime PP (mmHg)		0.28 (-0.09-0.65)	0.100	0.010	0.137	0.35 (0.08-0.63)	0.170	0.029	0.011	0.36 (-0.23-0.95)	0.080	0.006	0.235
Carotid-femoral PWV (m/s)		3.14 (-0.83-7.11)	0.099	0.010	0.120	0.22 (-2.77-3.21)	0.009	0.000	0.886	1.05 (-5.19-7.29)	0.021	0.000	0.740
Carotid-radial PWV (m/s)		-0.88 (-3.51-1.75)	-0.042	0.002	0.510	1.08 (-0.88-3.05)	0.069	0.005	0.279	-2.16 (-6.27-1.96)	-0.065	0.004	0.303
Central SBP (mmHg)	**PWV**	0.39 (0.14-0.65)	0.187	0.035	**0.003**	0.36 (0.17-0.55)	0.227	0.051	**<0.001**	0.48 (0.07-0.89)	0.144	0.021	0.022
Central DBP (mmHg)		0.19 (-0.31-0.69)	0.048	0.002	0.452	0.16 (-0.22-0.53)	0.051	0.003	0.418	0.73 (-0.05-1.52)	0.116	0.013	0.066
Central PP (mmHg)		0.37 (0.10-0.64)	0.169	0.029	**0.007**	0.34 (0.14-0.54)	0.209	0.043	**<0.001**	0.30 (-0.13-0.73)	0.087	0.008	0.168

B indicates unstandardized beta; 95% CI, 95% confidence interval; R2, nonadjusted R Square. The unstandardized coefficients (and 95% CIs) have been multiplied by 1000, showing the change in micrometers.

Significant *p* values (<0.01) bolded. AT indicates adventitia thickness; SBP, systolic blood pressure; DBP, diastolic blood pressure; PP, pulse pressure; PWV, pulse wave velocity; BMI, body mass index.

Supplementary Table 2. Univariate linear regression results for children's arterial lumen diameters

		**Common carotid artery lumen diameter**				**Brachial artery lumen diameter**				**Radial artery lumen diameter**				**Femoral artery lumen diameter**			
		B (95% CI)	Standardized β	R^2	*p*	B (95% CI)	Standardized β	R^2	*p*	B (95% CI)	Standardized β	R^2	*p*	B (95% CI)	Standardized β	R^2	*p*
Sex (0 = female, 1 = male)	**Sex**	0.18 (0.07-0.29)	0.193	0.037	**0.002**	0.24 (0.16-0.0.32)	0.341	0.116	**<0.001**	0.11 (0.05-0.17)	0.234	0.055	**<0.001**	0.44 (0.29-0.59)	0.335	0.112	**<0.001**
Age (years)	**Age**	-0.01 (-0.06-0.04)	-0.021	0.000	0.728	0.12 (0.08-0.15)	0.371	0.138	**<0.001**	0.07 (0.04-0.09)	0.314	0.099	**<0.001**	0.23 (0.17-0.30)	0.394	0.155	**<0.001**
Body height (cm)	**Anthropometrics**	0.01 (0-0.01)	0.126	0.016	0.040	0.02 (0.02-0.02)	0.542	0.294	**<0.001**	0.01 (0.01-0.01)	0.381	0.145	**<0.001**	0.04 (0.03-0.05)	0.552	0.305	**<0.001**
Height z-score		0.09 (0.03-0.14)	0.196	0.038	**0.001**	0.14 (0.10-0.17)	0.399	0.159	**<0.001**	0.05 (0.02-0.08)	0.220	0.048	**<0.001**	0.24 (0.17-0.31)	0.387	0.150	**<0.001**
Body weight (kg)		0.01 (0-0.01)	0.217	0.047	**<0.001**	0.02 (0.01-0.02)	0.498	0.248	**<0.001**	0.01 (0.01-0.01)	0.396	0.157	**<0.001**	0.03 (0.02-0.03)	0.422	0.178	**<0.001**
Weight z-score (height)		0.10 (0.05-0.15)	0.220	0.048	**<0.001**	0.08 (0.04-0.12)	0.236	0.056	**<0.001**	0.05 (0.03-0.08)	0.229	0.053	**<0.001**	0.09 (0.02-0.01)	0.141	0.020	0.021
Weight z-score (age)		0.13 (0.07-0.18)	0.280	0.078	**<0.001**	0.14 (0.10-0.18)	0.415	0.172	**<0.001**	0.07 (0.04-0.10)	0.305	0.093	**<0.001**	0.22 (0.15-0.29)	0.346	0.120	**<0.001**
Body surface area (m^2)		0.47 (0.21-0.73)	0.213	0.045	**<0.001**	0.88 (0.71-1.06)	0.529	0.280	**<0.001**	0.46 (0.34-0.58)	0.411	0.169	**<0.001**	1.46 (1.13-1.79)	0.471	0.222	**<0.001**
Lean body mass (kg)		0.01 (0-0.02)	0.179	0.032	**0.003**	0.03 (0.03-0.04)	0.597	0.356	**<0.001**	0.02 (0.01-0.02)	0.433	0.188	**<0.001**	0.06 (0.05-0.07)	0.573	0.328	**<0.001**
Skeletal muscle mass (kg)		0.02 (0.01-0.03)	0.176	0.031	**0.004**	0.05 (0.04-0.06)	0.598	0.358	**<0.001**	0.03 (0.02-0.03)	0.435	0.189	**<0.001**	0.09 (0.08-0.11)	0.574	0.330	**<0.001**
Head circumference (cm)		0.11 (0.08-0.14)	0.417	0.174	**<0.001**	0.08 (0.06-0.10)	0.390	0.152	**<0.001**	0.04 (0.03-0.06)	0.312	0.097	**<0.001**	0.16 (0.12-0.20)	0.424	0.180	**<0.001**
Thoracic circumference (cm)		0.02 (0.01-0.03)	0.312	0.098	**<0.001**	0.02 (0.02-0.03)	0.444	0.197	**<0.001**	0.01 (0.01-0.02)	0.377	0.142	**<0.001**	0.03 (0.02-0.04)	0.329	0.108	**<0.001**
Hip circumference (cm)		0.01 (0-0.02)	0.198	0.039	**0.001**	0.02 (0.01-0.02)	0.428	0.183	**<0.001**	0.01 (0.01-0.01)	0.349	0.122	**<0.001**	0.03 (0.02-0.04)	0.389	0.151	**<0.001**
Brachial circumference (cm)		0.04 (0.02-0.05)	0.249	0.062	**<0.001**	0.04 (0.03-0.05)	0.353	0.125	**<0.001**	0.02 (0.02-0.03)	0.316	0.100	**<0.001**	0.06 (0.03-0.08)	0.271	0.074	**<0.001**
Antebrachial circumference (cm)		0.07 (0.04-0.09)	0.316	0.100	**<0.001**	0.06 (0.04-0.08)	0.371	0.138	**<0.001**	0.04 (0.02-0.05)	0.319	0.102	**<0.001**	0.10 (0.06-0.13)	0.321	0.103	**<0.001**
Arm length (cm)		0.02 (0.01-0.04)	0.190	0.036	**0.002**	0.05 (0.04-0.06)	0.490	0.240	**<0.001**	0.02 (0.02-0.03)	0.368	0.135	**<0.001**	0.08 (0.06-0.10)	0.467	0.218	**<0.001**
Thigh circumference (cm)		0.02 (0.01-0.03)	0.213	0.045	**<0.001**	0.02 (0.01-0.03)	0.348	0.121	**<0.001**	0.01 (0.01-0.02)	0.309	0.095	**<0.001**	0.03 (0.02-0.05)	0.300	0.090	**<0.001**
Calf circumference (cm)		0.04 (0.02-0.05)	0.256	0.065	**<0.001**	0.05 (0.03-0.06)	0.426	0.182	**<0.001**	0.03 (0.02-0.03)	0.348	0.121	**<0.001**	0.07 (0.05-0.10)	0.378	0.143	**<0.001**
Leg length (cm)		0 (0-0.01)	0.064	0.004	0.309	0.02 (0.02-0.03)	0.456	0.208	**<0.001**	0.01 (0.01-0.01)	0.280	0.078	**<0.001**	0.04 (0.03-0.05)	0.393	0.154	**<0.001**
Waist-hip ratio (no unit)	**Adiposity**	1.18 (0.19-2.16)	0.143	0.021	0.019	1.19 (0.44-1.94)	0.190	0.036	**0.002**	0.71 (0.20-1.21)	0.168	0.028	**0.006**	1.53 (0.13-2.93)	0.131	0.017	0.032
BMI (kg/m^2)		0.03 (0.02-0.05)	0.231	0.053	**<0.001**	0.04 (0.03-0.05)	0.349	0.122	**<0.001**	0.02 (0.01-0.03)	0.308	0.095	**<0.001**	0.05 (0.03-0.07)	0.246	0.060	**<0.001**
BMI z-score		0.11 (0.06-0.16)	0.255	0.065	**<0.001**	0.11 (0.07-0.15)	0.318	0.101	**<0.001**	0.06 (0.04-0.09)	0.275	0.076	**<0.001**	0.15 (0.07-0.22)	0.234	0.055	**<0.001**
Fat mass (kg)		0.01 (0.01-0.02)	0.190	0.036	**0.002**	0.02 (0.01-0.02)	0.258	0.066	**<0.001**	0.01 (0.01-0.01)	0.243	0.059	**<0.001**	0.02 (0-0.03)	0.145	0.021	0.018
Fat percentage (%)		0.01 (0-0.02)	0.169	0.029	**0.006**	0.01 (0-0.01)	0.124	0.015	0.044	0 (0-0.01)	0.154	0.024	0.012	0 (-0.01-0.01)	0.025	0.001	0.682
24 hours SBP (mmHg)	**Ambulatory BP**	0 (-0.01-0)	-0.074	0.005	0.290	0 (0-0.01)	0.082	0.007	0.242	0 (-0.01-0)	-0.033	0.001	0.636	0 (-0.01-0.01)	0.042	0.002	0.547
24 hours DBP (mmHg)		0 (-0.01-0.01)	-0.020	0.000	0.775	-0.01 (-0.02-0)	-0.129	0.017	0.064	-0.01 (-0.01-0)	-0.115	0.013	0.099	-0.01 (-0.03-0.01)	-0.083	0.007	0.232
24 hours PP (mmHg)		-0.01 (-0.01-0)	-0.085	0.007	0.226	0.01 (0-0.02)	0.194	0.037	**0.005**	0 (0-0.01)	0.046	0.002	0.512	0.01 (0-0.02)	0.108	0.012	0.120
Daytime SBP (mmHg)		0 (-0.01-0)	-0.083	0.007	0.217	0 (0-0.01)	0.065	0.004	0.335	0 (-0.01-0)	-0.062	0.004	0.355	0 (-0.01-0.01)	0.042	0.002	0.533
Daytime DBP (mmHg)		0 (-0.01-0.01)	0.013	0.000	0.850	-0.01 (-0.01-0)	-0.084	0.007	0.211	0 (-0.01-0)	-0.100	0.010	0.134	0 (-0.02-0.01)	-0.039	0.002	0.562
Daytime PP (mmHg)		-0.01 (-0.02-0)	-0.114	0.013	0.088	0.01 (0-0.02)	0.155	0.024	0.020	0 (0-0.01)	0.015	0.000	0.821	0.01 (-0.01-0.02)	0.084	0.007	0.210
Nighttime SBP (mmHg)		0 (-0.01-0.01)	-0.019	0.000	0.781	0 (0-0.01)	0.064	0.004	0.345	0 (0-0)	0.055	0.003	0.415	0 (-0.01-0.01)	0.032	0.001	0.637
Nighttime DBP (mmHg)		0 (-0.01-0.01)	-0.044	0.002	0.516	-0.01 (-0.01-0)	-0.162	0.026	0.016	0 (-0.01-0)	-0.054	0.003	0.419	-0.01 (-0.02-0)	-0.109	0.012	0.106
Nighttime PP (mmHg)		0 (-0.01-0.01)	-0.032	0.001	0.632	0.01 (0-0.01)	0.197	0.039	**0.003**	0 (0-0.01)	0.099	0.010	0.142	0.01 (0-0.02)	0.125	0.016	0.064

B indicates unstandardized beta; 95% CI, 95% confidence interval; R2, nonadjusted R Square. Significant *p* values (<0.01) bolded. SBP indicates systolic blood pressure; DBP, diastolic blood pressure; PP, pulse pressure; BMI, body mass index.

Supplementary Table 3. Multiple linear regression models for children's arterial lumen diameters

**Vascular dimension**		**Predictor**	**Unstandardized β (95% CI)**	**Standardized β**	***p* value**	**Adjusted R2**	**Model *p* value**
**Brachial artery**							
Lumen diameter	**Model 1**	Constant	1.83 (1.66-2.00)			0.431	**<0.001**
		Pre-eclampsia exposed (0 = no, 1 = yes)	-0.06 (-0.13-0.01)	-0.082	0.082		
		Child sex (0 = female, 1 = male)	0.19 (0.12-0.26)	0.268	**<0.001**		
		Child lean body mass at follow-up (kg)	0.03 (0.03-0.04)	0.573	**<0.001**		
	**Model 2**	Constant	0.68 (0.20-1.17)			0.312	**<0.001**
		Pre-eclampsia exposed (0 = no, 1 = yes)	-0.03 (-0.11-0.05)	-0.039	0.452		
		Child sex (0 = female, 1 = male)	0.20 (0.12-0.27)	0.281	**<0.001**		
		Child arm length at follow-up (cm)	0.04 (0.03-0.05)	0.452	**<0.001**		
**Radial artery**							
Lumen diameter	**Model 1**	Constant	1.13 (1.00-1.26)			0.211	**<0.001**
		Pre-eclampsia exposed (0 = no, 1 = yes)	0 (-0.06-0.05)	-0.008	0.887		
		Child sex (0 = female, 1 = male)	0.09 (0.03-0.14)	0.181	**0.001**		
		Child lean body mass at follow-up (kg)	0.01 (0.01-0.02)	0.414	**<0.001**		
	**Model 2**	Constant	0.52 (0.16-0.89)			0.160	**<0.001**
		Pre-eclampsia exposed (0 = no, 1 = yes)	0.01 (-0.05-0.07)	0.015	0.797		
		Child sex (0 = female, 1 = male)	0.09 (0.03-0.14)	0.187	**0.001**		
		Child arm length at follow-up (cm)	0.02 (0.02-0.03)	0.341	**<0.001**		
**Femoral artery**							
Lumen diameter	**Model 1**	Constant	3.70 (3.38-4.02)			0.394	**<0.001**
		Pre-eclampsia exposed (0 = no, 1 = yes)	-0.02 (-0.16-0.11)	-0.017	0.719		
		Child sex (0 = female, 1 = male)	0.35 (0.23-0.48)	0.270	**<0.001**		
		Child lean body mass at follow-up (kg)	0.05 (0.04-0.06)	0.545	**<0.001**		
	**Model 2**	Constant	2.21 (1.39-3.03)			0.284	**<0.001**
		Pre-eclampsia exposed (0 = no, 1 = yes)	0.04 (-0.11-0.18)	0.025	0.638		
		Child sex (0 = female, 1 = male)	0.48 (0.35-0.62)	0.373	**<0.001**		
		Child leg length at follow-up (cm)	0.04 (0.03-0.05)	0.409	**<0.001**		

Significant results are bolded (*p* value <0.05). CI indicates confidence interval; BMI, body mass index.

1.1 Supplementary Figures

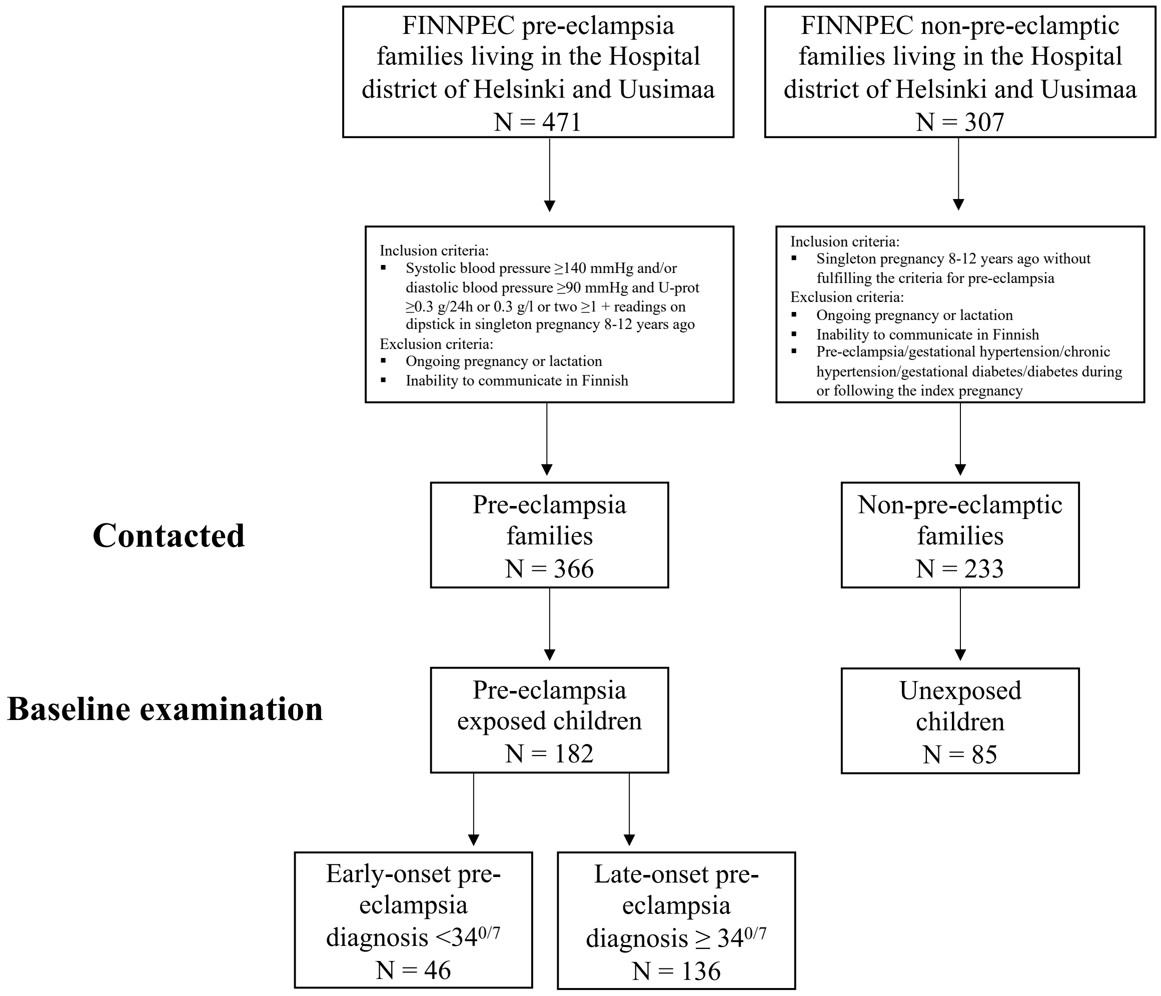

Supplementary Figure 1. Flowchart of the FINNCARE study.
